# Design, simplified cloning, and *in-silico* analysis of multisite small interfering RNA-targeting cassettes

**Published:** 2016-03

**Authors:** Bahram Baghban-Kohnehrouz, Shahnoush Nayeri

**Affiliations:** Department of Plant Breeding & Biotechnology, University of Tabriz, Tabriz, Iran

**Keywords:** Cloning strategy, Computational modeling, One-step PCR method, siRNA-targeting cassette

## Abstract

Multiple gene silencing is being required to target and tangle metabolic pathways in eukaryotes and researchers have to develop a subtle method for construction of RNA interference (RNAi) cassettes. Although, several vectors have been developed due to different screening and cloning strategies but still some potential limitations remain to be dissolved. Here, we worked out a simple cloning strategy to develop multisite small interfering RNA (siRNA) cassette from different genes by two cloning steps. In this method, effective siRNA sites in the target messenger RNAs (mRNAs) were determined using *in silico* analysis and consecutively arranged to reduce length of inverted repeats. Here, we used one-step (polymerase chain reaction) PCR by designed long primer sets covering the selected siRNA sites. Rapid screening, cost-effective and shorten procedure are advantages of this method compare to PCR classic cloning. Validity of constructs was confirmed by optimal centroid secondary structures with high stability in plants.

## INTRODUCTION

RNA interference (RNAi) technology has become a potentially powerful research tool for gene silencing applications like fighting against virus and parasite infections or functional analysis of gene(s) of interest [1-6]. Recently, several studies reported on gene expression by reducing the steady-state target messenger RNA (mRNA) levels without affecting the nuclear transcription or post-transcriptional gene silencing (PTGS) [7-14]. Although, phenomenon of RNAi has first discovered in nematode *Caenorhabditis elegans* but we are aware of it in plants, fungi, nematodes, protozoa, insects (*Drosophila* *melanogaster*) and vertebrates [15-16]. So far, RNAi has not been found in prokaryotes, most likely, it is a eukaryotic regulational mechanism [15]. RNAi-based gene silencing also protect the organism's genome from transposons and viruses. Furthermore, it could be a part of the defense system in plants [3, 17, 18]. The genetic and biochemical studies suggest that RNAi takes place in a very similar manner in many organisms and the enzymes involved in this process exhibit high homology cross species. Briefly, initial step is being connected with appearance of double-stranded RNA (dsRNA) molecules in the cell, which is perfectly homologous in sequence to the silenced gene. The dsRNA molecules were targeted by an RNase III-like enzyme (Dicer) producing either small interfering RNAs (siRNAs) or micro RNAs (miRNAs) of 20-24 nucleotides in length with additional characteristics, including 3'-hydroxyl termini, 2-nucleotide 3'-overhangs and 5'-phosphorelated termini. These short dsRNAs incorporated into multi-component nuclease complex known as RNA-induced silencing complex (RISC), where these RNAs function as sequence specific tags and target the silencing function to the homologous mRNAs [15]. The described process probably takes place in the cytoplasm. In plants and worms, amplification of the silencing signal and cell-to-cell RNAi spreading was observed. The RNA-dependent RNA polymerase (RdRp) enzyme has found in both plants and *C. elegans*. Moreover, it is responsible for the generation and amplification of siRNA into dsRNAs [15, 19].

According to recent studies related to RNAi mechanism, RNAi is triggered by any type of dsRNA molecule structures produced from transcripts of the endogenous transposons, viruses, viral satellites, viroids or transcripts of transgenes. The dsRNAs can also be introduced heterochromatic DNA that makes hairpin-like RNA secondary structure, experimentally [16]. Although in mammalian, nematodes and flies, si/miRNAs have been induced using either chemically synthesized siRNAs or vector-based short hairpin RNAs (shRNAs), in plants it is more efficiently achieved by specific expression cassettes that produce self-complementary hairpin-like RNA molecules [20-26]. However, realizing the importance of RNAi technology, leads to describing the different reports about the development of various vectors for the construction and expression of hairpin-like RNA constructs in plant cells. These include, pH/KANNIBAL, GATEWAY cloning system-based RNAi vectors like pHELLGATE and pIPK series has been widely used for generating intron-containing hairpin RNA (ihpRNA) constructs in plants [25, 27]. In these vectors, expression of an RNAi-inducing cassette will result in a dsRNA molecules composed of two distinct regions: a single-stranded loop, encoded by the spacer region and a double strand stem encoded by an inverted repeat [25]. With these properties, the efficiency of the RNAi-inducing cassette constructs account for the most determinant factor in generation of an effective high-throughput dsRNA molecule. 

According to several reports, the most RNAi-inducing cassette constructs were generated using polymerase chain reaction (PCR-based methods) and multiple restriction-ligation steps for cloning into desired RNAi-based vector, which is costly, tedious, and more time-consuming [20, 28-31]. In this work, we have identified efficient siRNA candidates from high-throughput study of siRNAs derived from transcript of plant gene of interest. To generate target repeats for making an effective dsRNA region, we have introduced a simple and effective one-step PCR method adopted using long primer sets, which were covered full regions of target mRNA with the number of highly potent siRNA content. Here, a simple strategy for rapid cloning of an inverted repeat for making multisite siRNA-targeting cassette is described.

## MATERIALS AND METHODS

**Plasmid materials** **: **The pTG19-T cloning vector (Vivantis, USA) derived from pTZ19-R vector (Accession no. in Genbank: Y14835.1) was used as a plasmid backbone.

**Determination of multiple presumed-siRNA sites in the target mRNAs:** So far, a number of experimental rules on siRNA duplex features have frequently reported. These include the asymmetry rules for siRNA duplex ends, high A/U content at the 5ʹ-end of the antisense strand, high G/C content at the 5ʹ-end of sense strand, 30-50% GC content, thermodynamic properties in term of the secondary structures and accessibilities of siRNA and target mRNA of gene(s) of interest [32-37]. The pssRNAit web server was used to *in silico *identified efficient siRNAs candidates in the gene(s) of interest (Http://plantgrn.noble.org/pssRNAit/). 

**Primer designing for One-step PCR: **The specific forward and reverse primers designed to conduct one-step PCR for synthesis of target sequences from desired genes (Table 1). These genes were α/β-gliadin (JX141486), γ-gliadin (FJ006593) and ω-gliadin (KF412584) multigenes from *Triticum aestivum* [10], small glutamin-rich tetratricopeptide gene (*SGT1) *(AY899199) from *Nicotinia bentamiana* [29], putrescine N-methyl transferase2 (*PMT2)* (AF126809) from *Nicotinia tabacum* [9] and fatty acid desaturase2-1 (*FAD2-1)* (AY954300) from *Glycine max* [13]. The primer sets of 50nt-long designed using Oligo version 7.56 analyzer software. The restriction sites of *Kpn*I, *Nde*I and *Xba*I, *Pst*I added at the 5'-ends of forward and reverse primers for product I and product II, respectively. To quarantine the base complementation at the 3'-end and efficient annealing of primers, the obtained primer sequences was checked using Oligo version 7.56 analyzer software. One-step synthesis reaction was carried out in a 25µl volume at 50ºC, consisting of 800nM of each 50-mers primers and 12.5 µl of 2X PCR Master Mix (Vivantis, USA; cat. no. PR8252C). The reaction was performed in only one extension step for 1 min at 72 ºC. The one-step amplificants were loaded using 1.2% agarose gel containing 20000X Red Safe™ (Intron biotechnology, USA; cat. no. 21141) 5% (v/v) and subjected to electrophoresis at 85 volts. The gels were photographed by gel imaging Quantum ST4 (Vilber Lourmat, France).

**Cloning of amplified multisite siRNA-targeting cassette: **The one-step PCR product I was purified by gel purification kit (Bioneer, South Korea; cat. no. K-3035). Then it was cloned into linear pTG19-T cloning vector by *T4* DNA ligase (200u/µl, Vivantis). The ligation reaction mixture was used in transformation of *Escherichia coli* strain DH5α competent cells. Following recovery of bacteria on antibiotic-free Lurai berthani (LB)-liquid culture (Miller), the cells were plated on LB-agar medium containing ampicillin (100µg/ml), 5-bromo-4-chloro-3-indolyl-β-D-galactopyranoside (X-gal) (100µg/ml) and Isopropyl β-D-1-thiogalactopyranoside (IPTG) (1mM) to screen the recombinants by blue/white system. DNA plasmids of white colonies were extracted from LB-liquid culture by plasmid extraction kit (Bioneer, South Korea; cat. no. K-3111). The inverted fragment (Product II) was sub-cloned into pTG-Direct by double digestion of *Pst*I/*Xba*I enzymes. The exact size of the siRNA cassette constructs were validated by *Pst*I restriction and sequencing. T7 promoter primer (TAA TAC GAC TCA CAT TAG GG) was used for sequencing.

**Table 1:** Sequences of the primer sets with 50-mer in length specialized for six genes of interest

**Gene name**	**Sequence of interest (Accession no.)**	**Position of SiRNA**	**Product no.**	**Primer sequence (5'→ 3')**
α-gliadin	JX141486	220-283	I	**F**:AGGGTACCTTGTATTGCAACAACACAGCAT**AGCGTATGGAAGCTCACAAG**
				**R**:AGCATATGTGGTAAGTACTTTGTTGCAAAA**CTTGTGAGCTTCCATACGCT**
			II	**F**:AGTCTAGATTGTATTGCAACAACACAGCAT**AGCGTATGGAAGCTCACAAG**
				**R**:AGCTGCAGTGGTAAGTACTTTGTTGCAAAA**CTTGTGAGCTTCCATACGCT**
ω-gliadin	KF412584	105-168	I	**F**:ACGGTACCTCCCATCAACAACAACCATTTC**CACAGCAGCCATATCCACAA**
				**R**:ATCATATGGTTGCTGTGATGGATATGGTTG**TTGTGGATATGGCTGCTGTG**
			II	**F**:ACTCTAGATCCCATCAACAACAACCATTTC**CACAGCAGCCATATCCACAA**
				**R**:ATCTGCAGGTTGCTGTGATGGATATGGTTG**TTGTGGATATGGCTGCTGTG**
γ-gliadin	FJ006593	420-502	I	**F**:ATGGTACCGCCCCAACAACAATTTCCGCAG**CCCCAACAACCACAACAATC**
				**R**:ATCATATGCGGTTGTTGTTGTTGGGGGAAT**GATTGTTGTGGTTGTTGGGG**
			II	**F**:ATTCTAGAGCCCCAACAACAATTTCCGCAG**CCCCAACAACCACAACAATC**
				**R**:ATCTGCAGCGGTTGTTGTTGTTGGGGGAAT**GATTGTTGTGGTTGTTGGGG**
*FAD2-1*	AY954300	1016-1080	I	**F**:ATGGTACCCTCTAGGAAGGGCTGTTTCTCT**TCTCGTCACACTCACAATAG**
				**R**:ATCATATGAAGGCTAAATACATAGGCCACC**CTATTGTGAGTGTGACGAGA**
			II	**F**:ATTCTAGACTCTAGGAAGGGCTGTTTCTCT**TCTCGTCACACTCACAATAG**
				**R**:ATCTGCAGAAGGCTAAATACATAGGCCACC**CTATTGTGAGTGTGACGAGA**
*PMT2*	AF126809	1500-1875	I	**F**:ATGGTACCATCGGCGGAGGAATTGGTTTTA**CATTATTCGAAATGCTTCGT**
				**R**:ATCATATGCAATTTTTTCGATTGTAGGATA**ACGAAGCATTTCGAATAATG**
			II	**F**:ATTCTAGAATCGGCGGAGGAATTGGTTTTA**CATTATTCGAAATGCTTCGT**
				**R**:ATCTGCAGCAAAAAACGATTGTAGGATA**ACGAAGCATTTCGAATAATG**
*SGT1*	AY899199	43-120	I	F:AGGGTACCTGACGACCACTTTGAGCTCGCC**GTTGACCTTTACACTCAAGC**
				R:ATCATATGGTTCTTAGGAGTCATGGCAATT**GCTTGAGTGTAAAGGTCAAC**
			II	F:AGTCTAGATGACGACCACTTTGAGCTCGCC**GTTGACCTTTACACTCAAGC**
				R:ATCTGCAGGTTCTTAGGAGTCATGGCAATT**GCTTGAGTGTAAAGGTCAAC**

**Computational modeling of hairpin RNA (hpRNA) secondary structure:** The hpRNA secondary structures derived from resulted multisite siRNA-targeting cassettes were predicted using Srna module in Sfold program (version 2.2) (http://sfold.wadsworth.org/cgi-bin/srna.pl/).

## RESULTS AND DISCUSSION

**Figure 1:**
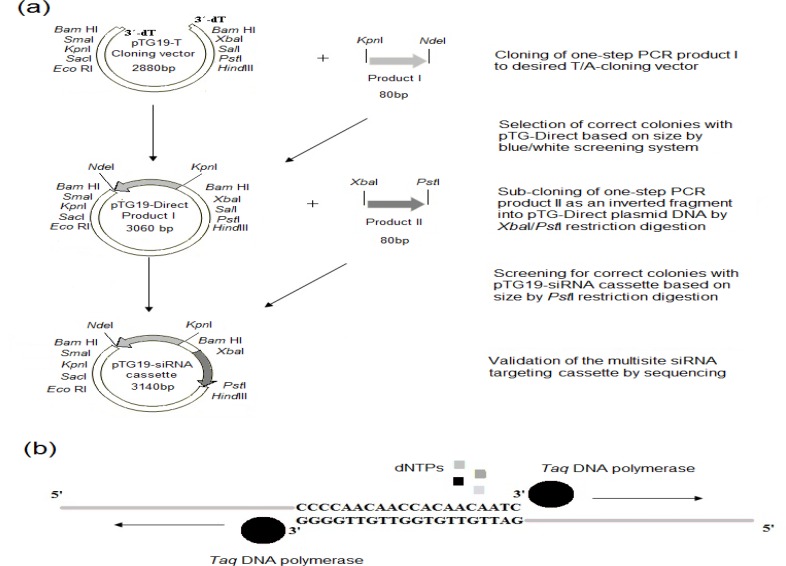
Schematic diagram of rapid and simple cloning strategy to obtain an efficient multisite siRNA-targeting cassette. (a) Strategy for cloning multisite siRNA-targeting cassette for gene of interest. (b) Diagram of Strategy for extending the inverted repeat. *Taq* DNA polymerase (circles) can extend in the presence of dNTPs (small colored squares) and PCR master mix the sense strand of two primers, which complemented 20nt-long at the 3´-end of both primers. The black arrows show the extending directions

Construction of RNAi vectors are currently used with several rounds of restriction/ligation steps and PCR-based methods, which are considered costly and time-consuming methods [25-30]. To adopt a simple strategy for making multisite siRNA cassette, the two-step procedure was carried out by a T/A cloning of one fragment (product I) followed by directional sub-cloning of its inverted orientation (product II). To achieve the spacer fragment without an additional cloning step, the 30bps fragment of cloning vector was used as the spacer between the inverted repeats. Total steps of the cloning strategy were illustrated in Fig 1a. Recently, one-step, zero-back ground ligation-independent cloning method (OZ-LIC) and pRNA Golden Gate (pRNA-GG) strategy were developed using one-step transformation. However, these methods have own potential limitations as the occasional presence of internal *Bsa*I site(s), several rounds of PCR reaction for amplifying the target repeats [27, 29]. Here, we propose a short cut method with one restriction-ligation step, comparatively less labor and cost-effective. 

Although, several studies reported the inverted repeats of 300-700 bp in length, the exact size of a dsRNA need to trigger RNAi in plants is still not entirely clear [24, 29]. However, the number of different potent siRNA sites within a target gene could be assuming as a critical point for triggering RNAi. Here, we elected totally six genes with and without intron containing from mono and dicot plants. The total number and nucleotide sequence of these siRNA sites was determined using pssRNAit server tool (Table 2). Consequently, three to four out of total 20 siRNA candidates were selected with 7-10 and 8-10 scores for off-target accessibility and efficiency values, respectively. In order to achieve a significant performance of dsRNA, the 3-4 highly potent different siRNA sites per gene were considered which consecutively arranged as the inverted repeats. This size reduction in the length of the previously reported target repeats is achievable by designing the specific long primer sets covering full region of siRNA sites (for more information see Table 1).

In recent decade, to develop a plant specific RNAi-based cassette with 300-700bp dsRNA region, PCR-based methods were commonly used for separately amplifying cassette fragments. Recently, a method based on one-step PCR process was developed in order to generate shRNA expression vectors for silencing mammalian genes [31]. However, presence of only a siRNA site in the dsRNA region of desired shRNA, designing of several primer sets to obtain multiple hpRNA for silencing one target gene and amplifying these cassettes by traditional PCR-based methods after one-step PCR process account for such limitations of this method. Here, to obtain 80bp dsRNA with the efficient consecutively arranged siRNA sites, specific long primer sets covered full region of target repeats were designed and used for a single cycle PCR reaction (Fig. 1b). Primer designing is extremely a critical step to achieve efficient amplificants and usually efforts have taken to use the minimum length (50-mers primer sets were designed for this work). 

**Figure 2:**
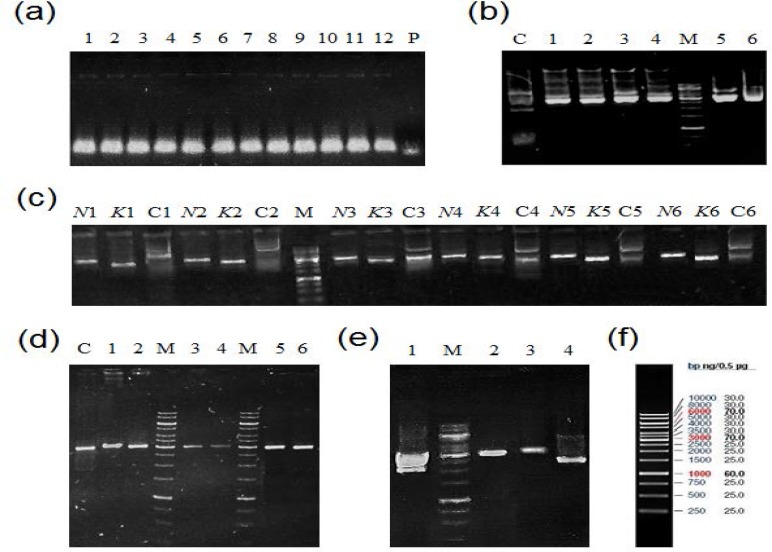
Screening of the inverted repeats retrieved from six genes of interest. (a) Confirmation of one-step PCR products I (Lanes 1-6) and products II (Lanes 7-12) in total six genes was illustrated, respectively. DNA concentrations and size of the one-step PCR products were determined using the 50-mer primer as a size control (lane P). (b) Screening of correct clones with product I inserts. The correct sizes of plasmid DNAs with product I for each cassette were validated by *Nde*I restriction digestion (Lanes 1-6) and compared with 1 kb DNA ladder (Lane M). The uncut-plasmid DNA as a control template was loaded in lane C. (c) Correct direction of product I inserts for each cassette was examined by *Kpn*I (Lanes *K*1-*K*6) and *Nde*I (Lanes *N*1-*N*6) restriction digestions and compared with 1 kb DNA gene ruler (Lane M). With correct orientation of insert, product I fragment excised from cloning vector. The uncut- plasmid DNAs (Lane C1-C6) validated the restriction digestion. (d) Validation of siRNA-targeting cassette constructs. The correct size of the DNA plasmids with double insert content have confirmed by *Pst*I restriction analysis (samples 1-6) and compared with gene ruler 1kb DNA ladder (Lane M) and empty vector (Lane C). (e) The plasmid DNA of pTG-Cassette (Lane 3) was distinguished based on size from plasmid DNA of pTG-Direct (Lane 2). The uncut-plasmid DNA of pTG-cassette and pTG-Direct were shown in Lanes 4 and 1, respectively. The correct size DNA concentration of pTG-siRNA Cassette was also determined by comparing to 1 kb DNA ladder (Lane M). (f) Illustration of gene ruler 1kb DNA ladder (Thermo Scientific Co., USA) with three sharp reference bands (6000, 3000 and 1000 bp) loaded on 0.8% agarose gel by Red Safe™ 5% (v/v

The amplificants of one-step PCR (80bp-long fragment in this work for both products I and II) were validated by 1.2% agarose gel electrophoresis using single primer as a size control (Fig. 2a). In the cloning of fragments, the correct colonies were screened out by standard blue/white screening system. On average, eight out of ten selected white colonies contained the recombinant DNA plasmid named as pTG-Direct with 3060bp long. The DNA plasmids with 80bps product I were confirmed by *Nde*I restriction digestion (Fig. 2b). A correct direction of insert was also distinguished from ones with opposite orientation by restriction analysis of plasmid DNA by *Kpn*I and *Nde*I restriction digestion (Fig. 2c).

**Table 2:** High-throughput siRNA candidates retrieved from template sequences of six genes of interest

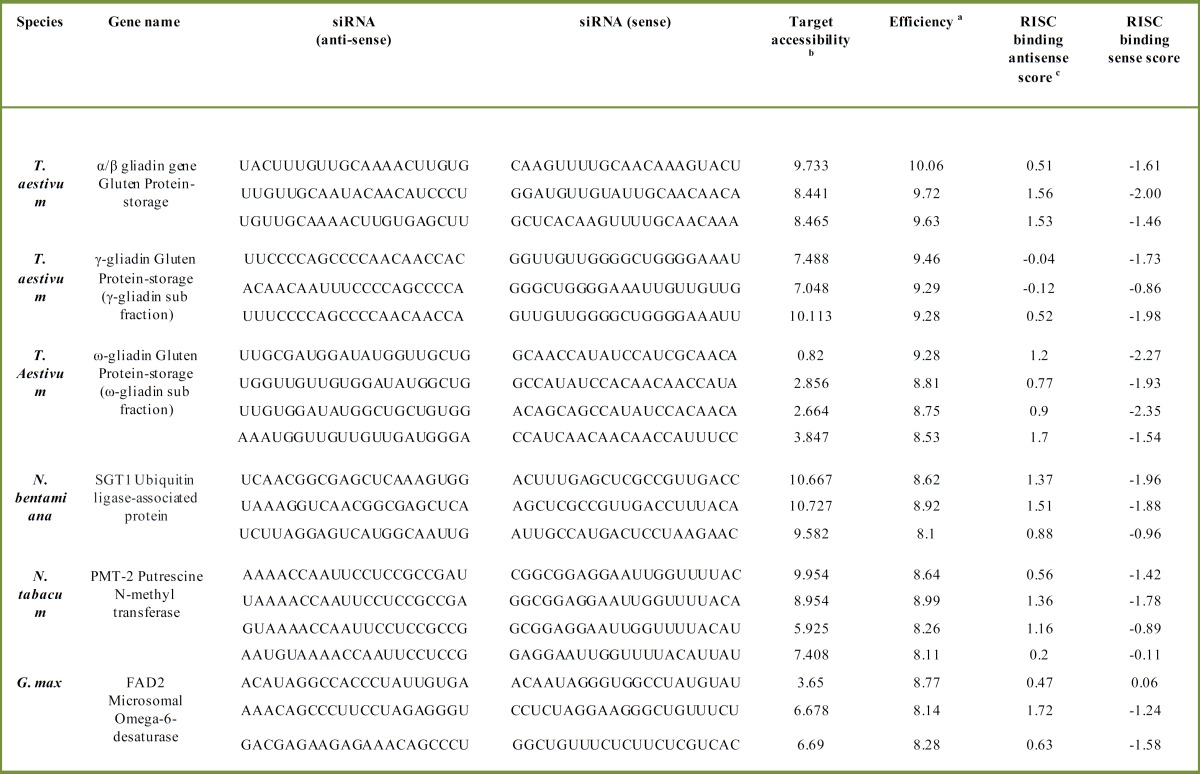

^a^Off-target accessibility or unpaired energy (UPE):P Pthe energy required to open mRNA secondary structure around target site is represented by UPE score in the range of 0-25 accessible scores. The less UPE score means the more possibility that siRNA is able to contact with target mRNA, which leads to the less off-target accessibility [38].

^b^siRNA efficiency: Efficiency denotes the effectiveness of designed siRNA to silence transcripts. The efficiency range can vary from 0-10, higher the value greater silencing of transcript [38].

The inverted *Xba*I/*Pst*I fragment was sub-cloned in pTG-Direct plasmid to develop siRNA cassette. In the resulted plasmid inverted repeat easily separated from direct fragment with a 30 bps spacer. For detailed information on siRNA cassette constructs from six genes of interests see fig 2(d). In fig 2(e), the plasmids containing the siRNA cassette named pTG-siRNA Cassette (3140bps in length, Lane 3) were distinguished from plasmids harboring only direct fragments (3060bps in length, Lane 2). The correct size and concentration of the all samples were determined by 1kb DNA ladder (Lane M). In Fig 2(f), the size and concentrations of the reference bands of 1kb DNA ladder are illustrated. Furthermore, the correct plasmid DNAs with siRNA cassettes was validated by sequencing analysis.

**Figure 3:**
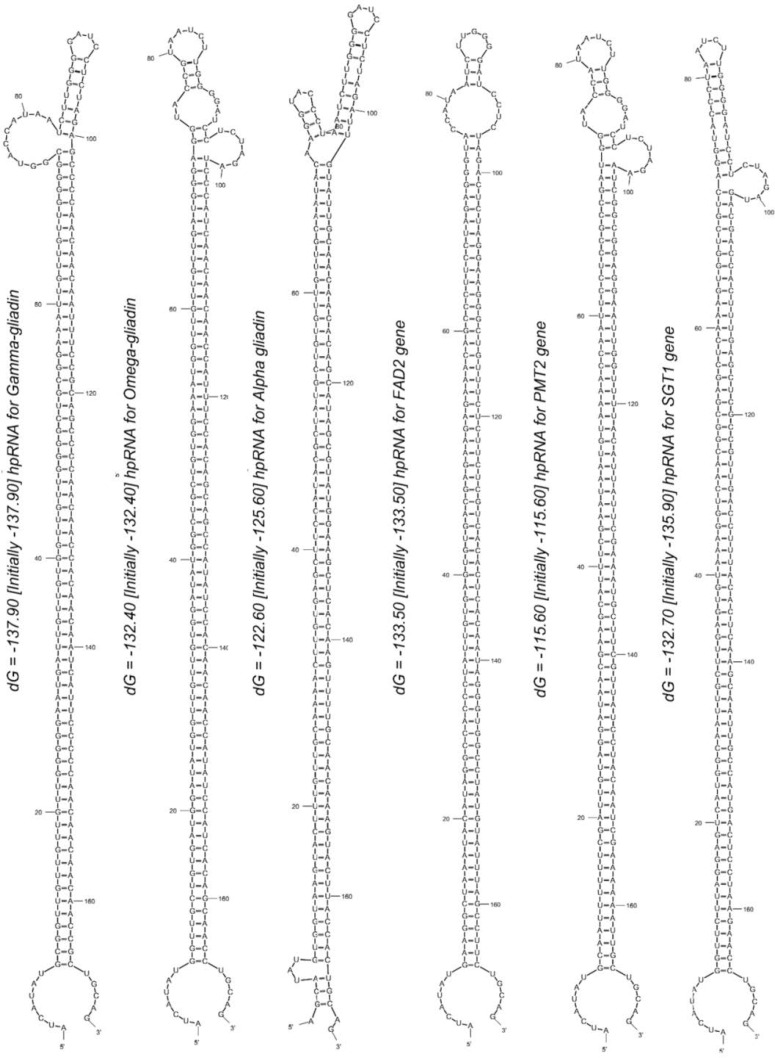
Illustration of the centroid structures for each six multisite siRNA targeting cassette of gene of interest with 156bp in length. The standard free energy (ΔG°) of optimum centroid structures for each gene of interest was shown

The hpRNA secondary structures of the all six siRNA cassettes were predicted by Srna module in Sfold program [39] and differences between centroid and minimum free energy (MFE) secondary structures derived from the siRNA targeting cassette constructs for six cassettes were shown in Table 3. We employ this approach with the aim of predicting the optimal hpRNA secondary structure with high stability for triggering RNAi mechanism in the target plant cell. According to reports, the ensemble centroid structure compared to MFE structure makes 30.0% fewer prediction errors and is closer to optimal secondary structure due to the lower average base-pair distance between the centroid structure and the sample [40-44]. Therefore, the ensemble centroid structure with minimum ∆Gº and base pair distance values was considered as the optimum hpRNA secondary structure for each six siRNA-targeting cassette. The diagram of the six optimum hpRNA structures illustrated that the sense and antisense strands of hpRNA stem could spontaneously stablish a full desired dsRNA region, which can be involved in Dicer cleavage and triggering RNAi process (Fig. 3).

**Table 3:** All properties of hpRNA secondary structures for six genes of interest and differences between MFE and Centroid structures

**Genes**	**MFE Structure ** b				**Centroid structure ** c			
	∆Gº	P-value	Boltzmann probability	base-pair distance	∆Gº	P-value d	Boltzmann probability	base-pair distance
**α/β-gliadin**	-125.60	0.819	0.313028	4.325	-122.60	Cluster1:0.819^a^ Cluster2:0.150 Cluster3:0.031	0.266145	3.499
**ω-gliadin**	-132.40	0.716	0.169359	6.337	-132.40	Cluster1:0.716 a Cluster2:0.284	0.0034487	6.325
**γ-gliadin**	-137.90	0.359	0.17994	7.178	-137.90	Cluster1:0.466 Cluster2:0.359 a Cluster3:0.175	0.0001679	5.654
**FAD2**	-133.30	0.536	0.0974788	10.153	-133.50	Cluster1:0.536 a Cluster2:0.464	0.0599123	8.269
**PMT2**	-115.60	0.654	0.13377	6.865	-115.60	Cluster1:0.654 a Cluster2:0.346	1.28788e	6.625
**SGT1**	-135.90	0.302	0.17118	3.87	-132.70	Cluster1:0.317 Cluster2:0.302 a Cluster3:0.155 Cluster4:0.116	0.17118	3.87

a MFE structure is located in the cluster of interest

b MFE structure: an RNA secondary structure based on minimum free energy probability

c Centroid structure: the structure in the entire structure ensemble that has the minimum total base-pair distance to the structures in the set.

d P-value: the base-pair probabilities computed from a statistical sample with a default size of 1000 structures for centroid structure of each cluster [39, 43].

In summary, with regard to the extensive applications of RNAi-based vectors in plant biotechnology research, we have developed a simple and cost-effective method for making a siRNA-targeting cassette containing efficient and consecutively arranged siRNAs from genes of interest with different gene structure in mono and dicot plants. In comparison of traditional PCR-based methods, rapid and cost-effective procedure account for such advantages of one-step PCR method. Furthermore, the optimal centroid structures with high stability in plant cells were achieved using computational modeling of the secondary structure of the cassettes.

## Notes

**Acknowledgements: **This work was financially supported by department of Plant Breeding and Biotechnology, Tabriz University, Tabriz, Iran.
